# Polyhydroxyalkanoate Production from Eucalyptus Bark’s Enzymatic Hydrolysate

**DOI:** 10.3390/ma17081773

**Published:** 2024-04-12

**Authors:** Thomas Rodrigues, Cristiana A. V. Torres, Susana Marques, Francisco Gírio, Filomena Freitas, Maria A. M. Reis

**Affiliations:** 1 Associate Laboratory i4HB, Institute for Health and Bioeconomy, School of Science and Technology, NOVA University Lisbon, 2829-516 Caparica, Portugal; ta.rodrigues@campus.fct.unl.pt (T.R.); c.torres@fct.unl.pt (C.A.V.T.); amr@fct.unl.pt (M.A.M.R.); 2 UCIBIO—Applied Molecular Biosciences Unit, Department of Chemistry, School of Science and Technology, NOVA University Lisbon, 2829-516 Caparica, Portugal; 3 Unidade de Bioenergia e Biorrefinarias, Laboratório Nacional de Energia e Geologia I.P., 2610-999 Lisboa, Portugal; susana.marques@lneg.pt (S.M.); francisco.girio@lneg.pt (F.G.)

**Keywords:** polyhydroxyalkanoates, lignocellulose, saccharification, hydrolysate, enzyme

## Abstract

In recent years, polyhydroxyalkanoates (PHAs) have gained notoriety because of their desirable properties that include proven biodegradability, biocompatibility, and thermal stability, which make them suitable alternatives to fossil-based polymers. However, the widespread use of PHAs is still challenging because of their production costs, which are greatly associated with the cultivation medium used for bacterial cultivation. In Portugal, one-quarter of the forest area is covered by *Eucalyptus globulus* wood, making its residues a cheap, abundant, and sustainable potential carbon source for biotechnological uses. In this work, eucalyptus bark was used as the sole feedstock for PHA production in a circular bioeconomic approach. Eucalyptus bark hydrolysate was obtained after enzymatic saccharification using Cellic^®^ CTec3, resulting in a sugar-rich solution containing glucose and xylose. Although with differing performances, several bacteria were able to grow and produce PHA with distinct compositions, using the enzymatic hydrolysate as the sole carbon source. *Pseudomonas citronellolis* NRRL B-2504 achieved a high cellular growth rate in bioreactor assays (24.4 ± 0.15 g/L) but presented a low accumulation of a medium-chain-length PHA (mcl-PHA) comprising the monomers hydroxydecanoate (HD, 65%), hydroxydodecanoate (HDd, 25%), and hydroxytetradecanoate (HTd, 14%). *Burkholderia thailandensis* E264, on the other hand, reached a lower cellular growth rate (8.87 ± 0.34 g/L) but showed a higher biopolymer accumulation, with a polyhydroxybutyrate (PHB) content in the cells of 12.3 wt.%. The new isolate, *Pseudomonas* sp., revealed that under nitrogen availability, it was able to reach a higher accumulation of the homopolymer PHB (31 wt.%). These results, although preliminary, demonstrate the suitability of eucalyptus bark’s enzymatic hydrolysate as a feedstock for PHA production, thus offering an exciting avenue for achieving sustainable and environmentally responsible plastic products from an undervalued forestry waste.

## 1. Introduction

Polyhydroxyalkanoates (PHAs) are well-known biopolymers that, because of their biodegradability, sustainability, and thermal stability, have emerged as promising and environmentally friendly alternatives for the replacement of fossil-fuel-derived polyesters (e.g., polyethylene terephthalate and polyamides) [1]. PHAs can find use, for instance, in medicine and pharmacy (e.g., medical textiles and disposable wipes) or be processed into biodegradable fibres for eco-friendly clothing or coatings [2]. However, their production cost is a critical factor that still affects their wider utilisation.

PHA synthesis relies on microbial cultivation, and the selection of suitable carbon sources plays an essential role in the reduction of production costs [3,4]. Taking that into consideration, the use of low-cost feedstocks, such as agricultural wastes, organic materials, and industrial byproducts, has been investigated for enhancing the economic viability of PHAs, as well as aligning it with the principles of the circular economy [3,4]. The recent research focus has demonstrated that the utilisation of residual biomass derived from agricultural and forest sources, consisting of lignocellulosic biomass (LCB), can lead to a substantial reduction of around 40–50% in substrate costs for microbial cultivation, thus contributing to the yield of PHAs at a more competitive price while also facilitating the valorisation of LCB resources [5,6].

The bioconversion of LCB, based on the so-called “sugar platform”, requires the prior hydrolysis of its constituent polysaccharides, i.e., cellulose and hemicellulose fractions, to obtain the respective monomers (mostly glucose and xylose). This step is advantageously accomplished by enzymatic hydrolysis because of this method’s higher intrinsic specificity, avoiding/reducing the formation of degradation products (possibly obtained from sugars—furan derivatives and weak acids—and lignin—phenolics), which would inhibit the subsequent biological process. Nevertheless, most LCBs are structurally rigid and compact, and, therefore, their polysaccharides are not easily accessible to enzymes. Thus, LCB, contrary to sugar and starch feedstocks, needs to be subjected to mechanical comminution and to pretreatment before the application of the enzymatic process to reduce the recalcitrance of the lignocellulose matrix by liberating it from lignin, reducing the cellulose crystallinity and increasing the porosity of the material [3,5,7]. Indeed, LCB pretreatment is the most technically difficult and one of the most expensive processing steps in LCB-to-fermentable-sugar conversion. LCB pretreatment can be accomplished by several technologies—biological, physical, chemical, or thermochemical—but most of them are not yet mature, requiring further development and demonstration before successful commercial implementation [8].

Owing to these challenges, up to this point, the commercial production of PHAs has relied on sugar-based cultivated feedstocks, such as food crops, molasses, whey, vegetable- and fruit-processing wastes, corn, palm, and vegetable oils [9,10], which can be easily broken down into individual monosaccharides (e.g., glucose) [5]. In the present work, non-catalysed steam explosion, a variant of hydrothermal (physicochemical) technology—solely applying saturated water vapor at high pressures, was used for biomass processing, bringing the advantage for generating low levels of inhibitory products. Steam explosion constitutes, in fact, one of the most well-developed pretreatment technologies that is already applied in several demonstration and first commercial second-generation ethanol units [8].

In Portugal, 25.8% (8.12 × 10^5^ ha) of the forest area is covered by *Eucalyptus globulus* wood [11]. This species ranks as the main feedstock in the pulp and paper industries, offering significant advantages because of its rapid growth and favourable chemical and anatomical properties, which are suited for pulping [12]. As a result, substantial eucalyptus-based residues are generated, including bark (around 0.5 Mton in 2017 in Portugal) [13]. This LCB material is primarily composed of polysaccharides (up to 60%), mainly based on glucose and xylose [11,13].

In this context, this study aimed to explore the valorisation of eucalyptus bark as a low-cost feedstock for PHA production. The pretreated eucalyptus bark residue was saccharified by enzymatic hydrolysis to yield a sugar-rich hydrolysate that was used for bacterial cultivation. Different PHA producers were screened for their ability to grow on the eucalyptus hydrolysate and synthesise PHA. The produced biopolymers were extracted from the biomass and characterised for their composition.

## 2. Materials and Methods

### 2.1. Feedstock Preparation

#### 2.1.1. Raw Material

Eucalyptus bark supplied by the pulp mill of Cacia (Aveiro, Portugal), from the Navigator Company, was used as the feedstock. When received, the eucalyptus bark was homogenised in a defined lot and stored in plastic containers at room temperature. Each lot was chemically characterised to determine the exact polysaccharide content for further use. The lignocellulosic raw material was gravimetrically analysed for water (by oven-drying at 105 °C to a constant weight) and ash (by ignition at 575 °C to a constant weight) contents. Polysaccharide (hemicellulose and cellulose) and lignin contents were assayed by means of quantitative hydrolysis with sulphuric acid in two stages (the first step with 72% (*w*/*w*) acid at 30 °C for 1 h and the second with 4% (*w*/*w*) acid for 1 h at 121 °C) based on the NREL/TP-510-42618 protocol [14]. The acid-insoluble residue was considered as Klason lignin after correction for the acid-insoluble ash. The acid-soluble lignin was determined in the corresponding filtrate by UV spectroscopy at 320 nm (NREL/TP-510-42618 protocol [14]). The quantification of the liberated monosaccharides and acetic acid was carried out by high-performance liquid chromatography (HPLC) using an Aminex HPX-87H (300 × 7.8 mm) column (Bio-Rad, Hercules, CA, USA) operating at 50 °C with 0.01 N H_2_SO_4_ as the mobile phase at a flow rate of 0.6 mL/min.

#### 2.1.2. Pretreatment

In the first step, eucalyptus bark was pretreated based on a proprietary non-catalysed steam explosion technology, without the addition of acids and using only high-pressure steam, developed by the company STEX^®^ (Aveiro, Portugal) in partnership with the National Laboratory of Energy and Geology (LNEG, Lisboa, Portugal). Steam explosion was carried out in a 320 L reactor coupled to a 4000 L blow tank, where the pretreated material was discharged by the sudden depressurisation of the reactor. After the two-step pretreatment at 205 °C (17.5 bar) for 10 and 3 min, respectively, as described in [15], the solid fraction collected by coarse filtration (85% of the pretreated biomass on an oven-dried basis) was washed with water at room temperature and then directly used for the enzymatic hydrolysis. Prior to the enzymatic hydrolysis, a sample of the pretreated eucalyptus bark was chemically characterised (adopting the same methodology as described for the raw material in Section 2.1.1). The collected liquid fraction was also characterised to complement the pretreatment monitoring. Monosaccharides (glucose, xylose, and arabinose), acetic and formic acids, and sugar degradation products were directly analysed by HPLC. The concentration of the oligosaccharides was determined based on the increase in the concentration of the monosaccharides, as analysed by HPLC, after post-hydrolysis under standard conditions (corresponding to the addition of sulphuric acid at an overall concentration of 4% (*w*/*w*) during 60 min at 121 °C). An Agilent 1260 series (Waldbronn, Germany) HPLC equipped with refractive index (RI) and diode array (DAD) detectors, the latter being set at a fixed wavelength of 280 nm, was used for furan (5-hydroxymethyl-2-furfural (5-HMF) and furfural) quantification.

#### 2.1.3. Enzymatic Hydrolysis

A commercial cellulolytic enzymatic (also containing highly active xylanases) cocktail from Novozymes A/S (Bagsværd, Denmark)—Cellic^®^ CTec3 HS—was used for the enzymatic hydrolysis. The eucalyptus bark hydrolysate was obtained after 48 h of enzymatic saccharification of the pretreated solid residue. The procedure was performed at 50 °C at an initial solid concentration (oven-dried (o.d.) basis) of 175 g/L by applying Cellic^®^ CTec3 HS at an enzymatic load of 3% (*w*/*w* o.d. solids). This suspension was incubated in a 600 L stirred-tank reactor, and the resulting hydrolysate was centrifuged (at 12,000× *g* for 15 min at 50 °C) to remove the unreacted solids and then appropriately stored (frozen) until use. The hydrolysate was analysed by HPLC (directly and post hydrolysis, as described above), and its ammonium concentration was determined by colorimetry using a flow-segmented analyser (Skalar 5100, Skalar Analytical, Breda, The Netherlands).

### 2.2. PHA Production

#### 2.2.1. Microorganisms and Media

*Pseudomonas oleovorans* NRRL B-14682, *Pseudomonas oleovorans* NRRL B-14683, *Pseudomonas citronellolis* NRRL B-2504, a new isolate of *Pseudomonas* sp., and *Burkholderia thailandensis* E264 were used in this study. Luria–Bertani (LB) broth (Bacto tryptone (Fisher Scientific, Hampton, NH, USA), 10 g/L; yeast extract (Biolife, Bothell, WA, USA), 5 g/L; sodium chloride (Fisher Scientific, 99.5%), 10 g/L, pH 6.8) was utilised for the inoculum preparation, while Medium E* supplemented with the enzymatic hydrolysate was used for the cultivation assays. The composition (per litre) of the Medium E* was as follows: (NH_4_)_2_HPO_4_, 1.1 g; K_2_HPO_4_, 5.8 g; KH_2_PO_4_, 3.7 g; 10 mL of a 100 mM MgSO_4_ solution; and 1 mL of a micronutrient solution [9]. The composition of the micronutrient solution (per litre of 1 N HCl) was as follows: FeSO_4_⋅7H_2_O (Sigma-Aldrich, St. Louis, MO, USA), 2.78 g; MnCl_2_⋅4H_2_O (Sigma-Aldrich), 1.98 g; CoSO_4_⋅7H_2_O (Sigma-Aldrich), 2.81 g; CaCl_2_⋅2H_2_O, 1.67 g; CuCl_2_⋅2H_2_O (Sigma-Aldrich), 0.17 g; and ZnSO_4_⋅7H_2_O (Sigma-Aldrich), 0.29 g. The pH of the media was set to 7.0 by the addition of NaOH prior to autoclaving at 121 °C and 1 bar for 20 min.

#### 2.2.2. Inoculum Preparation

The cultures were reactivated from cryopreserved vials (at −80 °C in 20% (*v*/*v*) glycerol) by plating them onto CHROMagar™ Orientation agar plates (Paris, France) and incubating them for 48 h at 30 °C. For the pre-inoculum preparation, a single colony was collected from the agar plate, inoculated in 20 mL of LB broth in a 100 mL Erlenmeyer flask, and incubated in an orbital shaker (at 200 rpm) for 24 h at 30 °C. Afterwards, 20 mL of the pre-culture was inoculated in 200 mL of Medium E* supplemented with the enzymatic hydrolysate in a 500 mL baffled shake flask and grown for 72 h under the same conditions as the pre-inoculum to obtain the inoculum for the cultivation assays.

#### 2.2.3. Shake Flask Assays

The shake flask assays consisted of the cultivation of each bacterial strain in 200 mL of Medium E* supplemented with the enzymatic hydrolysate (at an initial sugar concentration of 31.4 g/L) in 500 mL baffled shake flasks in an orbital shaker (at 200 rpm) at 30 °C for ~50 h. Samples (3 mL) were periodically collected from the shake flasks for monitoring the cellular growth by measuring their optical density at a wavelength of 600 nm. At the end of the assays, the cultivation broth was centrifuged (at 9000× *g* for 15 min) and used for the quantification of the cellular dry weight (CDW) and the PHA content in the cells and the determination of the PHA composition. All the experiments were performed in duplicate.

#### 2.2.4. Bioreactor Cultivation

The experiments were carried out by cultivating *Pseudomonas citronellolis* NRRL B-2504, *Burkholderia thailandensis* E264, and *Pseudomonas* sp. in 2 L bioreactor runs (BioStat B-Plus, Sartorius, Germany) using Medium E* supplemented with the hydrolysate (at an initial sugar concentration of 31.4 g/L). The bioreactors were inoculated with a 10% (*v*/*v*) inoculum (200 mL), prepared as described above, and operated in the batch mode. The pH and temperature were controlled at 7.0 ± 0.1 and 30 ± 0.1 °C, respectively. The pH was controlled by the automatic addition of 2 M HCl and 5 M NaOH (Merck, Darmstadt, Germany). The airflow was kept constant (2 SLPM, standard litres per minute) throughout the runs. The concentration of the dissolved oxygen (DO) decreased from full saturation at the beginning of the runs to 30% of the air saturation, the value at which it was controlled by the automatic adjustment of the stirrer speed between 200 and 2000 rpm. Antifoam A (Sigma-Aldrich) was added automatically to avoid foam formation. Samples (12 mL) were collected from the bioreactor and centrifuged (at 9000× *g* for 15 min) for cellular separation. The cell-free supernatant was preserved at −20 °C for the sugar, acid, and ammonium quantifications, while the cellular pellets were used for the quantification of the CDW and determination of the PHA content and composition.

#### 2.2.5. Analytical Techniques

The cellular pellet obtained by the centrifugation of the culture broth was washed with deionised water and lyophilised. The CDW was gravimetrically determined by weighing the lyophilised pellets. The composition and content of the PHA within the cells were determined by gas chromatography (GC), following the methanolysis method described by Rebocho et al. [16]. Briefly, ~5 mg of the dried cellular pellet was hydrolysed with 2 mL of 20% (*v*/*v*) sulphuric acid (Chem-Lab, 95–97%, Zedelgem, Belgium) in methanol (Fisher Chemical, HPLC grade, Waltham, MA, USA) and 2 mL of benzoic acid in chloroform (1 g/L) (Sigma-Aldrich, HPLC grade) at 100 °C for 4 h. The calibration curve for 3-hydroxyhexanoate (3HHx), 3-hydroxyoctanoate (3HO), 3-hydroxydecanoate (3HD), 3-hydroxydodecanoate (3HDd), and 3-hydroxytetradecanoate (3HTd) was generated using an in-house-produced mcl-PHA with the following composition, as validated by GC-MS (GC—Agilent 6890N (Santa Clara, CA, USA); MS—Thermo DSQ (West Palm Beach, FL, USA)): 3 mol% 3HHx, 17 mol% 3HO, 57 mol% 3HD, 11 mol% 3HDd, and 12 mol% 3HTd at concentrations ranging from 0.1 to 2.0 g/L [17]. The co-polymer poly(3-hydroxybutyrate-co-3-hydroxyvalerate) (PHBHV, 9/1, Sigma-Aldrich) was used to obtain a calibration curve for 3-hydrohybutyrate (3HB) and 3-hydroxyvalerate (3HV) at concentrations between 0.05 and 1 g/L and using benzoic acid as an internal standard. The methyl esters were analysed in a Restek column (Crossbond, Stabilwax, Bellafonte, PA, USA) at a constant pressure, using helium as a carrier gas, through splitless injection. The oven temperature was programmed at 20 °C/min until 100 °C, 3 °C/min until 155 °C, and 20 °C/min until 220 °C.

The sugar concentrations were determined by HPLC, using a CarboPac PA10 column (Dionex, Sunnyvale, CA, USA) equipped with an amperometric detector. The analysis was conducted using 4 mM NaOH as the eluent at 30 °C and a flow rate of 0.9 mL/min. A standard solution composed of anhydrous 99% D-(+)-glucose (Fisher Chemical) and 99% D-(+)-xylose (Sigma-Aldrich) was prepared in deionised water in a concentration range from 1 to 100 ppm. The ammonium concentration was determined by colorimetry as described above.

The acid concentrations were analysed by HPLC, using a VARIAN Metacarb 87H column (BioRad, Heracles, CA, USA) coupled to a refractive index (RI) detector. The analysis was conducted at 50 °C utilising H_2_SO_4_ as the eluent at a flow rate of 0.6 mL/min. A standard solution composed of glacial acetic acid (Fisher Chemical) and formic acid (Sigma-Aldrich) was prepared in deionised water in a concentration range from 0.01 to 1 g/L.

All the analyses were performed in triplicate.

#### 2.2.6. Calculations

The maximum specific growth rate (*µ*_max_, h^−1^) was calculated from the linear regression slope of the exponential phase of Ln X_t_ versus time, where X_t_ represents the rest of the biomass (without PHA) at time t (h) as follows:

X_t_ = CDW_t_ − PHA_t_ (1)

where CDW_t_ (g/L) and PHA_t_ (g/L) represent the CDW and the concentration of the PHA at time t (h), respectively. The concentration of the PHA is determined from the polymer content within the bacterial cells on a dry basis (wt.%). The volumetric productivity (r_p_, g/(L·h)) was determined by dividing the final concentration of the PHA (g/L) by the total cultivation time (Δt, h).

The polymer yield on a substrate basis (Y_P/S_, g_PHA_/g_sugar_) was determined using Equation (2) as follows:

Y_P/S_ = ΔP/ΔS (2)

where ΔP is the overall produced PHA (g/L), and ΔS (g/L) is the total sugar consumed over the cultivation.

The growth yield on a substrate basis, Y_X/S_ (g_X_/g_sugar_), was calculated using Equation (3) as follows:

Y_X/S_ = ΔX/ΔS (3)

where ΔX (g/L) is the rest of the biomass, and ΔS (g/L) is the total sugar consumed over the assay.

### 2.3. PHA Extraction

Upon the completion of the assays, the cultivation broth was centrifugated (at 13,000× *g* for 20 min), and the biomass pellet was lyophilised. The PHA was extracted from the dried biomass (10 g) by Soxhlet extraction with chloroform (250 mL) at 80 °C for 48 h followed by PHA purification through precipitation in ice-cold ethanol (1:10 *v*/*v*), as described by Pereira et al. [18]. The polymers were dried at room temperature in a fume hood and kept in closed vessels.

## 3. Results and Discussion

### 3.1. Feedstock Characterisation

The compositions of the pretreated biomass and the initial raw materials, allowing for the mass balances of the pretreatment, are shown in Table 1, demonstrating that the cellulose (glucan) was almost completely retained in the solid, whereas there was an extensive (58.2%) solubilisation of the xylan. The collected liquid fraction consisted of glucose, 0.1; xylose, 2.2; gluco-oligosaccharides, 0.8; xylo-oligosaccharides, 3.5; acetyl groups, 0.1; acetic acid, 5.4; hydroxymethylfurfural, 0.3; furfural, 1.3 g/L. This composition is aligned with the significant xylan solubilisation and degradation.

**Figure 1:**
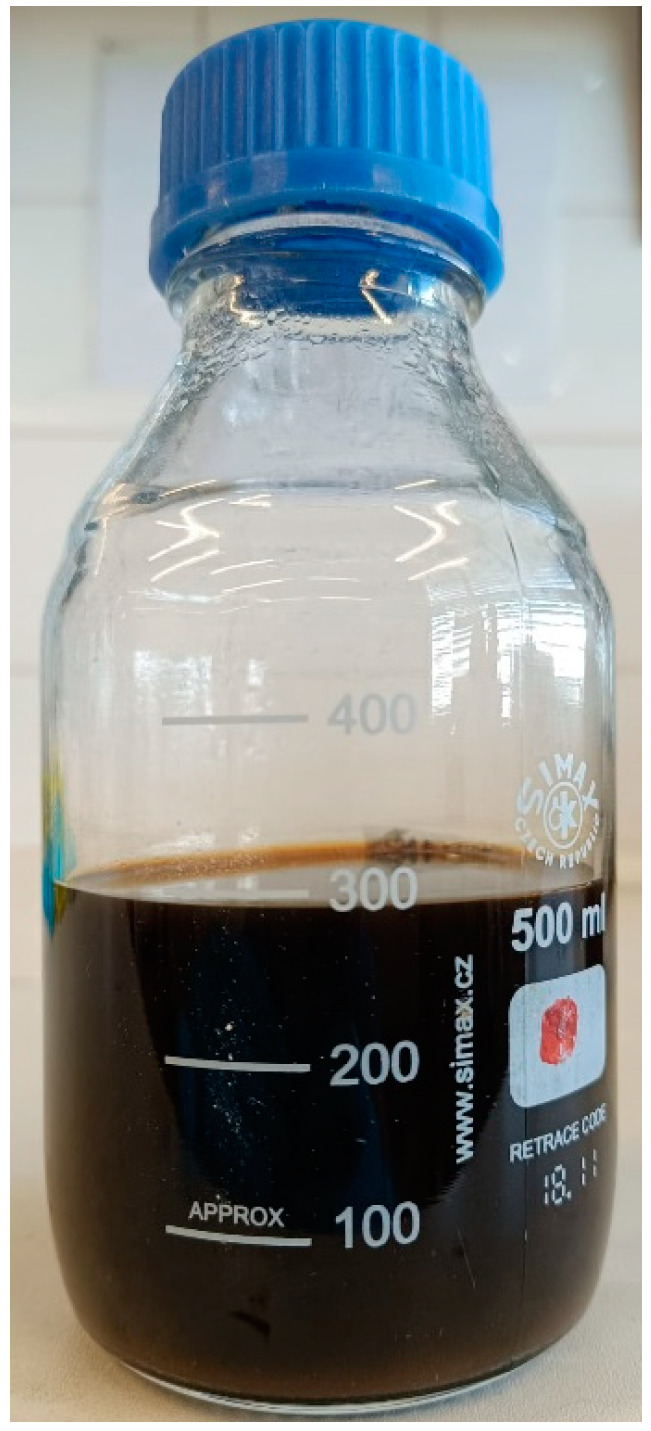
Image of the hydrolysate obtained by the enzymatic hydrolysis of the eucalyptus bark.

As can be observed in Figure 1, the final hydrolysate was a dark, opaque, non-viscous liquid, with no visible suspended particles (even after centrifugation). The hydrolysate was characterised in terms of sugar and degradation product, acid, and ammonium concentrations. It mostly comprised glucose (68.6 ± 0.7 g/L) and xylose (8.47 ± 0.17 g/L), corresponding to enzymatic hydrolysis yields of 92.3% and 61.8%, respectively, for cellulose and xylan conversion to their constituent monosaccharides. This composition is common among lignocellulosic hydrolysates because they usually consist of 60–70% glucose and 30–40% xylose [7]. In addition, gluco- (2.2 g/L) and xylo-oligosaccharides (0.3 g/L) were present in the hydrolysate, resulting from the incomplete hydrolysis of polysaccharides. Moreover, the hydrolysate contained formic acid (0.494 ± 0.021 g/L) and acetic acid (1.17 ± 0.038 g/L) together with vestigial concentrations of 5-HMF and furfural, which could act as an inhibitor of bacterial cellular growth [19]. In lignocellulosic hydrolysates, the presence of aliphatic acids is common because the hydrolysis of hemicellulose acetyl groups generates acetic acid, while the thermochemical degradation of polysaccharides gives rise to formic and levulinic acids [20] and, further, to furans. Also, the hydrolysate presented 0.58 ± 0.022 g/L of ammonium, which could be used by the bacteria as a nitrogen source for cellular growth [21].

### 3.2. Shake Flask Screening

**Figure 2:**
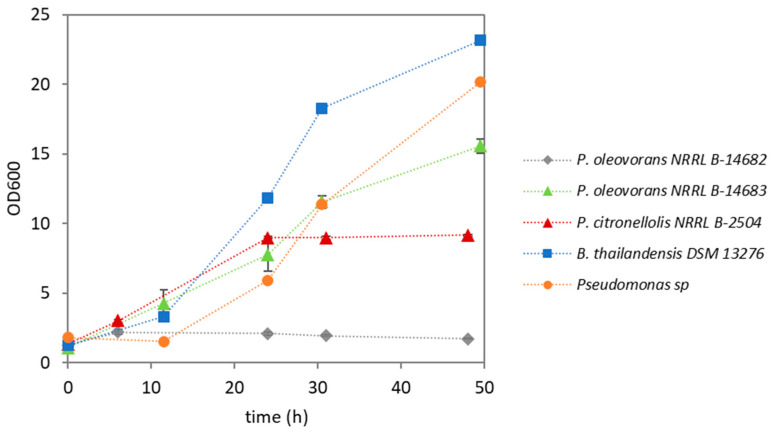
Cellular growth profile, based on the optical density measured at a wavelength of 600 nm (OD600), for the tested bacterial strains.

In this study, different PHA-producing bacteria were cultivated using the eucalyptus bark enzymatic hydrolysate as the sole feedstock. As shown by the profiles of the optical density measured at 600 nm (OD600), all the strains were able to grow in the hydrolysate-supplemented medium (Figure 2), although with differing performances in terms of cellular growth and PHA accumulation (Table 2). Except for *Ps. oleovorans* NRRL B-14682, for which OD600 increased only slightly, for all the other tested strains, there was a significant increase in OD600 within 25–30 h of cultivation (Figure 2).

As presented in Table 2, at the end of assay, *Pseudomonas* sp. showed, among the tested bacterial strains, the highest cellular growth rate, reaching a CDW of 7.37 ± 0.58 g/L. This value is higher than most of those reported in the literature (1.9–5.1 g/L) for strains grown in different agriculture and forestry wastes/byproducts (e.g., grape pomace and rice straw) subjected to saccharification by either acidic or enzymatic treatments [8,15]. *Pseudomonas* sp. was also the strain attaining the highest polymer accumulation in the cells (38.9 ± 4.1 wt.%), producing the homopolymer PHB (Table 2). This strain was isolated from a lignocellulosic hydrolysate that was contaminated during storage, and given its ability to grow in the feedstock, it was included in this study.

*B. thailandensis* DSM 13276, on the other hand, also showed promising PHA accumulation, as shown by the polymer content in the biomass (25.4 ± 2.0 wt.%) for a CDW of 6.30 ± 0.50 g/L (Table 2). These values are higher than those reported by Blunt et al. [22] for the same strain grown in xylose as the sole carbon source (16.4%) but lower than the values reached for glucose (40.5%) or glucose/xylose mixtures (64%). *B. thailandensis* DSM 13276 produced the homopolymer PHB in accordance with the literature that reported the ability of this species to synthesise this type of PHA [23].

As expected, based on the OD600 profile (Figure 2), *Ps. oleovorans* NRRL B-14682 had a low cellular growth rate, with a CDW of 1.31 ± 0.19 g/L at the end of the run. No polymer was detected in the biomass, probably because of the low cellular growth rate observed for this strain under the tested conditions (Table 2). *Ps. oleovorans* NRRL B-14683, on the other hand, reached a CDW of 4.05 ± 0.41 g/L, with a PHA content in the biomass of 1.1 ± 0.1 wt.% (Table 2). The produced polymer was composed of 3HB monomers.

Regarding *Ps. citronellolis* NRRL B-2504, despite its lower cellular growth rate (CDW = 2.60 ± 0.20 g/L) upon cultivation in the hydrolysate-supplemented medium, it produced an mcl-PHA, reaching a polymer content in the biomass of 3.6 ± 0.1 wt.% (Table 2). Nevertheless, although low, the CDW attained by this culture is within the range of values reported for this strain (2.83–3.68 g/L) grown in a grape pomace aqueous extract in 500 mL shake flasks [24]. The polymer was an mcl-PHA composed of 3HD (56%) and 3HO (28%), with minor contents of HTd (7%), HDd (6%), and HHx (3%).

Given these preliminary results, *Pseudomonas* sp. and *B. thailandensis*, the two best PHB producers, were selected to carry out bioreactor assays under controlled conditions of pH, temperature, DO concentration, aeration, and agitation to validate these strains’ abilities to utilise the eucalyptus hydrolysate as the sole feedstock for cellular growth and PHA accumulation. *P. citronellolis*, despite its lower cellular growth rate and polymer accumulation, was also selected for the bioreactor assays because it was the only mcl-PHA-producing strain among the tested bacteria.

### 3.3. Bioreactor Assays

#### 3.3.1. Bioreactor Cultivation of *Pseudomonas citronellolis* NRRL B-2504

**Figure 3:**
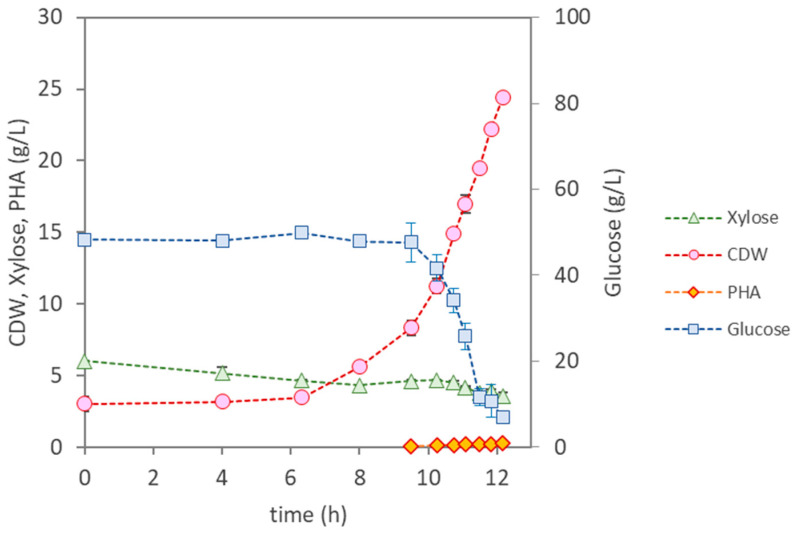
Cultivation profile for *Pseudomonas citronellolis* NRRL B-2504, using the eucalyptus hydrolysate as the sole feedstock.

In a batch bioreactor assay (Figure 3), *Ps. citronellolis* had a lag phase of about 6 h, after which it grew at a maximum specific cellular growth rate of 0.37 h^−1^, reaching a CDW of 24.4 ± 0.15 g/L (Table 3) after 12 h of cultivation. A total intake of 41 g/L of glucose and 1.6 g/L of xylose were observed, mostly for cellular growth, as the polymer content in the biomass was very low (1%) compared with the value reported (30%) by Rebocho et al. [16], with a lower CDW (4 g/L), using apple pulp waste as the sole carbon source. The corresponding growth yield was 0.49 g_X_/g_sugar_ (Table 3). This culture was also able to consume the acetic acid (1.95 g/L) and the formic acid (0.55 g/L) present in the hydrolysate. Thus, under the tested cultivation conditions, *Ps. citronellolis* proved to be able to efficiently use the nutrients in the hydrolysate for cellular growth. However, PHA synthesis was impaired by the exhaustion of the carbon sources. Longer cultivation runs in a fed-batch mode could guarantee the substrate’s availability and effective PHA accumulation.

#### 3.3.2. Bioreactor Cultivation of *Burkholderia thailandensis* DSM 13276

**Figure 4:**
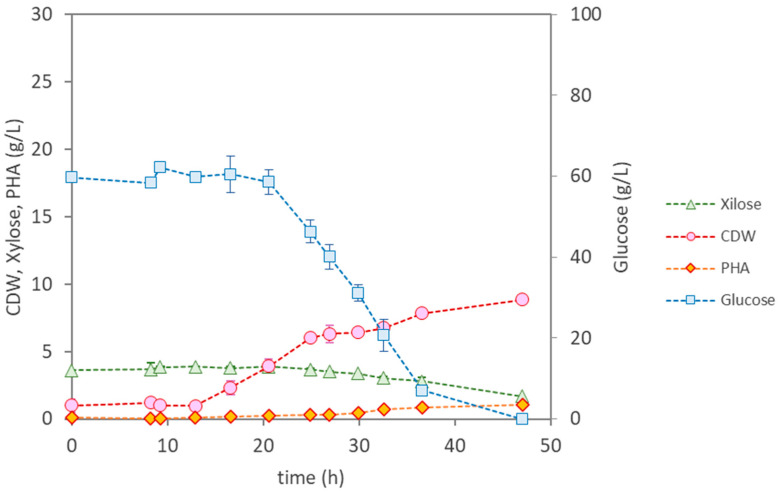
Cultivation profile for *Burkholderia thailandensis* DSM 13276, using the eucalyptus hydrolysate as the sole feedstock.

*B. thailandensis*, on the other hand, had a longer lag phase (around 13 h) (Figure 4) and grew at a lower maximum specific cellular growth rate (0.13 h^−1^) (Table 3). It attained a maximum CDW of 6.32 ± 0.13 g/L at around 27 h of cultivation when the ammonium was depleted. PHA synthesis was initiated then, reaching a content in the biomass of 12% by the end of the cultivation (47 h). This corresponds to a final PHA production of 1.10 g/L and an overall volumetric productivity of 0.023 g_PHA_/(L·h). Total concentrations of 59.7 g/L of glucose and 1.9 g/L of xylose were consumed during the run, corresponding to growth and product yields of 0.13 g_X_/g_sugar_ and 0.018 g_PHA_/g_sugar_, respectively (Table 3). These values are lower than those reported by Blunt et al. [22] (40.5 wt.%), with glucose as the sole carbon source, for this strain. Interestingly, *B. thailandensis* synthesised a PHA comprising 3HV (1.4%), which, even at low contents, is known to confer interesting properties to the biopolymer, namely, increased flexibility, compared to the homopolymer PHB.

Although a low polymer content in the biomass was achieved by *B. thailandensis*, these results show the abilities of this strain to effectively assimilate the sugars present in the feedstock and accumulate PHA, revealing this to be a promising culture for valorising eucalyptus hydrolysate into value-added products.

#### 3.3.3. Bioreactor Cultivation of *Pseudomonas* sp.

**Figure 5:**
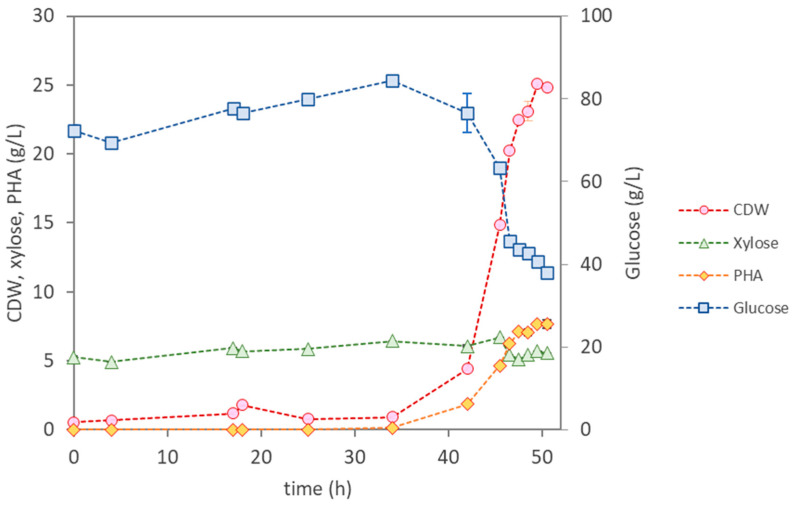
Cultivation profile for the isolate *Pseudomonas* sp., using the eucalyptus hydrolysate as the sole feedstock.

Two assays were performed with *Pseudomonas* sp., in which different initial ammonium concentrations were tested, namely, 0.82 g/L (assay P1) and 1.42 g/L (assay P2) (Table 3). In both assays, a rather long lag phase was observed (30–34 h), after which the culture grew at identical maximum specific cellular growth rates (0.22 h^−1^). As expected, a significantly higher CDW value (24.83 ± 0.04 g/L) was attained in assay P2 (Figure 5) compared to assay P1 (11.30 ± 0.19 g/L) because of the higher nitrogen-source availability. Moreover, assay P2 also showed a higher polymer production rate, with the biomass attaining a PHA content of 31%, while a content of 18.2% was reached in assay P1 (Table 3).

These results might be explained by the fact that a higher glucose intake (46.6 g/L) was observed in assay P2 compared to assay P1 (22.1 g/L). For P1, that corresponds to growth and product yields of 0.24 g_X_/g_sugar_ and 0.27 g_PHA_/g_sugar_, respectively (Table 3). However, for P2, 46.6 g/L of glucose and 0.85 g/L of xylose were consumed (growth and product yields of 0.35 g_X_/g_sugar_ and 0.16 g_PHA_/g_sugar_, respectively), which suggest that the increase in the ammonium concentration led to higher sugar consumption for cellular growth and lower for PHA production. Concomitantly, a higher PHA production was reached in assay P2 (7.7 g/L), representing an overall volumetric productivity of 0.46 g_PHA_/(L·h). No xylose was consumed in assay P1, and only 0.85 g/L of the available 10.2 g/L were consumed in assay P2, suggesting this species’ preference for glucose intake over xylose [23,24], and the glucose was not depleted during the assay.

#### 3.3.4. Overall Assessment

Comparing the results obtained in the bioreactor cultivation runs with data reported for several bacteria grown in different LCB hydrolysates (Table 4), it is clear that PHA accumulation by *Ps. citronellolis*, *B. thailandensis*, and the isolate *Pseudomonas* sp. fell short for most of them. Despite that, *B. thailandensis* and the isolate *Pseudomonas* sp. still reached values within the range reported for scl-PHA producers (8–89%) (Table 4). Moreover, such results were obtained under non-optimised conditions and, thus, open the possibility of further improvement of the bioprocesses to optimise the conversion of the hydrolysate’s sugars to PHA and maximise the polymer content in the cells. Even for *Ps. citronellolis*, for which PHA accumulation was not significant, this bioprocess can be optimised by implementing it, for example, in the fed-batch mode, similar to that reported by Silva [23] for the conversion of the grape pomace hydrolysate to mcl-PHA by the same strain.

The eucalyptus hydrolysate is a complex mixture, containing not only glucose and xylose but also organic acids and other degradation products that can compromise the cultures’ ability to be utilised as a feedstock. Nevertheless, this study was successful in identifying these three strains as being able to grow in the eucalyptus hydrolysate, representing the start of novel routes for the valorisation of this LCB waste.

## 4. Conclusions

The eucalyptus bark’s enzymatic hydrolysate was successfully employed for PHA production by all the tested bacterial strains, but with different performances, including *Ps. oleovorans* NRRL B-14682 and NRRL B-14683, *Ps. citronellolis* NRRL B-2504, *B. thailandensis* E264, and *Pseudomonas* sp. *Ps. citronellolis* NRRL B-2504 was the only mcl-PHA-producing strain but did not attain a high PHA accumulation. *Pseudomonas* sp. was the most promising strain among the PHB producers, achieving the highest bacterial growth and PHA production rates when the ammonium concentration was increased, demonstrating its positive impact on PHA synthesis. These results revealed that the eucalyptus bark’s enzymatic hydrolysate is a potential alternative to synthetic media to promote PHA production at a lower cost while also contributing to a circular economy.

## Figures and Tables

**Table 1:** Chemical characterisation (expressed as a mass percentage of the oven-dried (o.d.) weight) of eucalyptus bark as received and after pretreatment (solid fraction recovered after steam explosion).

Component	As Received	Pretreated	
	(g/100 g_o.d._)	(g/100 g_o.d._)	(Percentage Recovered)
Glucan	33.3 ± 0.4	38.1 ± 0.6	97.3
Xylan	12.2 ± 0.2	6.0 ± 0.3	41.8
Arabinan	1.2 ± 0.1	0.0	0
Acetyl Groups	3.2 ± 0.2	0.4 ± 0.1	-
Klason Lignin	23.2 ± 0.6	27.5 ± 0.9	-
Acid-soluble Lignin	0.5 ± 0.1	0.1 ± 0.0	-
Ash	8.5 ± 0.4	10.5 ± 0.5	-
Others	17.9	17.4	-

**Table 2:** CDWs and PHA accumulations of the different tested PHA-producing strains using lignocellulose-based biomass hydrolysate as the sole feedstock in shake flasks (n.d.—not detected).

Strain	CDW (g/L)	PHA (wt.%)	Type of PHA
*Pseudomonas oleovorans* NRRL B-14682	1.31 ± 0.19	n.d.	---
*Pseudomonas oleovorans* NRRL B-14683	4.05 ± 0.42	1.1 ± 0.1	PHB
*Pseudomonas citronellolis* NRRL B-2504	2.60 ± 0.20	3.6 ± 0.1	mcl-PHA
*Pseudomonas* sp.	7.37 ± 0.58	38.9 ± 4.1	PHB
*Burkholderia thailandensis* DSM 13276	6.30 ± 0.50	25.4 ± 2.0	PHB

**Table 3:** Kinetic and stoichiometric parameters of the different tested PHA-producing strains upon bioreactor cultivation with the hydrolysate as the sole feedstock.

Parameter	*P. citronellolis* NRRL B-2504	*B. thailandensis* E264	*Pseudomonas* sp.	
			Assay P1	Assay P2
Cultivation Time (h)	12	47	49	50
*µ*_max_ (h^−1^)	0.37	0.13	0.22	0.22
CDW (g/L)	24.4 ± 0.15	8.87 ± 0.34	11.30 ± 0.19	24.83 ± 0.04
PHA (wt.%)	<1	12.3	18	31
PHA (g/L)	0.25	1.10	2.10	7.70
rPHA (g_PHA_/(L·h))	-	0.023	0.110	0.150
[Glucose] Uptake (g/L)	41.0	59.7	22.1	46.6
[Xylose] Uptake (g/L)	1.6	1.9	0	0.85
Y_PHA/S_ (g_PHA_/g_sugar_)	0.0058	0.018	0.27	0.16
Y_X/S_ (g_X_/g_sugar_)	0.49	0.13	0.24	0.35
PHA Composition	HD (65%); HDd (25%); HTd (14%)	HB (98.6%); HV (1.4%)	HB (100%)	HB (100%)

**Table 4:** PHA production from different bacteria using LCB-based hydrolysates as sole feedstocks for cultivation.

Strain	LCB	Hydrolysis	Scale	Cultivation Mode	Type of PHA	CDW (g/L)	PHA (%)	Ref.
*Pseudomonas citronellolis* NRR B-2504	Grape Pomace	Acidic	Shake Flask	Batch	mcl-PHA	2.83–3.68	9.1	[23]
			Bioreactor	Fed Batch		9.3	14	
*Halomonas halophila* *CCM 3662*	Grape Pomace	Enzymatic	Shake Flask	Batch	PHB	5.1	72	[8]
			Bioreactor			3.1	57	
*Pseudomonas cepacia*	Trembling Aspen	Enzymatic	Shake Flask	Batch	PHB	2.6	60	[15]
*Burkholderia* sp. *F24*	Sugarcane Bagasse	Acidic	Shake Flask	Batch	PHB	25.0	49	[24]
*Burkholderia sacchari* DSM17165	Wheat Straw	Enzymatic	Bioreactor	Fed Batch	PHB	100–140	72	[25]
*Cupriavidus necator* A-04	Pineapple Core	Acidic	Shake Flask/ Bioreactor	Batch	PHB	5.3–13.6	13.2–56.6	[26]
*Bacillus firmus*	Rice Straw	Acidic	Shake Flask	Batch	PHB	1.9	89	[27]
*Bacillus* MG12	Sugarcane	Acidic	Shake Flask	Batch	PHBHV	2.89	8	[28]

## Data Availability

Data will be made available upon request.
